# Histological Image Classification Between Follicular Lymphoma and Reactive Lymphoid Tissue Using Deep Learning and Explainable Artificial Intelligence (XAI)

**DOI:** 10.3390/cancers17152428

**Published:** 2025-07-22

**Authors:** Joaquim Carreras, Haruka Ikoma, Yara Yukie Kikuti, Shunsuke Nagase, Atsushi Ito, Makoto Orita, Sakura Tomita, Yuki Tanigaki, Naoya Nakamura, Yohei Masugi

**Affiliations:** Department of Pathology, School of Medicine, Tokai University, 143 Shimokasuya, Isehara 259-1193, Kanagawa, Japan; ih3822@tokai.ac.jp (H.I.); yy-kikuti@tokai.ac.jp (Y.Y.K.); ito.atsushi.s@tokai.ac.jp (A.I.); orita.makoto.k@tokai.ac.jp (M.O.); sakura.t@tokai.ac.jp (S.T.); tanigaki.yuki.s@tokai.ac.jp (Y.T.); naoya@tokai.ac.jp (N.N.); masugi@tokai.ac.jp (Y.M.)

**Keywords:** follicular lymphoma, follicular hyperplasia, reactive lymphoid tissue, deep learning, convolutional neural network, artificial intelligence, explainable artificial intelligence

## Abstract

**Background/Objectives**: The major question that confronts a pathologist when evaluating a lymph node biopsy is whether the process is benign or malignant, and the differential diagnosis between follicular lymphoma and reactive lymphoid tissue can be challenging. **Methods**: This study designed a convolutional neural network based on ResNet architecture to classify a large series of 221 cases, including 177 follicular lymphoma and 44 reactive lymphoid tissue/lymphoid hyperplasia, which were stained with hematoxylin and eosin (H&E). Explainable artificial intelligence (XAI) methods were used for interpretability. **Results**: The series included 1,004,509 follicular lymphoma and 490,506 reactive lymphoid tissue image-patches at 224 × 244 × 3, and was partitioned into training (70%), validation (10%), and testing (20%) sets. The performance of the training (training and validation sets) had an accuracy of 99.81%. In the testing set, the performance metrics achieved an accuracy of 99.80% at the image-patch level for follicular lymphoma. The other performance parameters were precision (99.8%), recall (99.8%), false positive rate (0.35%), specificity (99.7%), and F1 score (99.9%). Interpretability was analyzed using three methods: grad-CAM, image LIME, and occlusion sensitivity. Additionally, hybrid partitioning was performed to avoid information leakage using a patient-level independent validation set that confirmed high classification performance. **Conclusions**: Narrow artificial intelligence (AI) can perform differential diagnosis between follicular lymphoma and reactive lymphoma tissue, but it is task-specific and operates within limited constraints. The trained ResNet convolutional neural network (CNN) may be used as transfer learning for larger series of cases and lymphoma diagnoses in the future.

## Simple Summary

The major question that confronts a pathologist when evaluating a lymph node biopsy is whether the process is benign or malignant, and the differential diagnosis between follicular lymphoma and reactive lymphoid tissue can be challenging. This study used deep learning and explainable artificial intelligence to predict follicular lymphoma and reactive lymphoid tissue using hematoxylin and eosin histological images in a large series of 221 cases.

## 1. Introduction

The major question that confronts a pathologist when evaluating a lymph node biopsy is whether the process is benign or malignant [1].

The latest World Health Organization and International Consensus Classifications include more than 80 types of mature lymphoid neoplasm that contain B-cell, T-cell, and Hodgkin lymphomas. The lymphoma subtypes are defined according to their morphology, immunophenotype, cell derivation or cell of origin, clinical characteristics, and molecular features [2,3,4,5,6,7,8,9].

The broad category of mature B-cell neoplasms include Chronic lymphocytic leukemia (CLL)/small lymphocytic lymphoma (SLL), Lymphoplasmacytic lymphoma (LPL), Monoclonal gammopathies, Plasma cell neoplasms, Hairy cell leukemia, Marginal zone lymphoma (MZL), Follicular lymphoma (“classic FL”, FL grade 3B, FL with unusual features with blastoid or predominantly diffuse growth pattern), Mantle cell lymphoma (MCL), Diffuse large B cell lymphoma (DLBCL), High-grade B cell lymphomas (Burkitt lymphoma, high-grade B cell lymphoma with *MYC* and *BCL2* and/or *BCL6* rearrangement, high-grade B cell lymphoma NOS, and aggressive B cell lymphomas with 11q aberration), Hodgkin lymphoma (Classic HL, and Nodular lymphocyte-predominant HL (NLPHL) [2,3,4,5,6,7,8,9].

Follicular lymphoma (FL) is defined as a neoplasia of B lymphocytes of the germinal center (typically both centrocytes and centroblasts) that usually depicts a partially follicular (nodular) pattern [6,10]. It is clinically indolent and characteristically harbors the t(14;18)(q23;q32)/*BCL2*::*IGH*. FL represents around 40% of adult lymphomas in Western countries and 20% worldwide [11,12]. The median age of presentation is 65 years with generalized lymphadenopathy, splenomegaly, often asymptomatic, and bone marrow infiltration in 40% of the cases [11,12]. Widespread disseminated disease is usually present at baseline, but patients are typically asymptomatic, except for lymphadenopathy. The architectural pattern of FL shows enlarged lymph nodes with effacement by uniform, closely packed, and evenly distributed neoplastic follicles [11]. The diagnosis is primarily made based on lymph node biopsy evaluation. Tumor cells express immunoglobulin light chain and are positive for CD20, CD10, BCL6, and BCL2 but negative for CD5 and CD23 immunohistochemical markers [6,10]. The main differential diagnoses of FL are follicular hyperplasia, progressive transformation of germinal centers, other small B-cell lymphomas (small lymphocytic lymphoma, mantle cell lymphoma, and marginal zone lymphoma), and Hodgkin lymphoma (nodular lymphocyte-predominant Hodgkin lymphoma and nodular sclerosis classic Hodgkin lymphoma) [6,10].

Diffuse areas in FL are defined as an area that lacks evidence of neoplastic follicles but contains a mixture of centrocytes and centroblasts. Classically, the WHO classification [10,11] recognized three patterns of grade 1 to 2 FL: follicular (>75% follicular), follicular and diffuse 25–75%), predominantly diffuse (>25% follicular), and diffuse (0% follicular). In low-grade (1 to 2) FL, diffuse areas are not prognostically relevant. However, diffuse areas composed predominantly of centroblasts (grade 3) are diagnosed as diffuse large B-cell lymphoma. Purely diffuse pattern FL is rare, and a significant subset may fall under the new provisional entity defined as *BCL2*-R-negative, CD23+ follicle center lymphoma that is characterized by *STAT6* mutations [6].

The establishment of FL grade was based on the idea that clinically aggressive cases had an increased number of centroblasts (large nucleolated cells) [10,11]. The grading was the following: ≤5 centroblasts/high-power field (hpf), grade 1; 6–15 centroblasts/hpf, grade 2; and >15 centroblasts/hpf, grade 3. In the WHO 4th edition, grades 1 and 2 were combined into the “low-grade” category [10,11]. Grade 3 is divided into A (centrocytes still present) and B (solid sheets of centroblasts). The presence of *BCL2* rearrangements and expression of CD10 favor grade 3A [6]. In the 5th edition of WHO, FL grade 3B goes under the name of Follicular Large B-cell Lymphoma [13].

According to the architectural histologic pattern, reactive lymphadenopathies [14,15] are grouped into four major categories: follicular/nodular, sinus, interfollicular or mixed, and diffuse. However, multiple nodal compartments may be involved in a single process, and variation exists from case to case. The classification of reactive lymphadenopathies is the following: (1) Follicular and nodular patterns (follicular hyperplasia and autoimmune disorders); (2) Predominant sinus patterns, sinus histiocytosis (Whipple disease [16]); (3) Interfollicular or mixed pattern (Kimura disease [17], systemic lupus erythematosus, and Kikuchi disease [18,19]); (4) Diffuse pattern (infectious mononucleosis and cytomegalovirus infection) [6,10,11,12,13] (Table 1).

Follicular hyperplasia is characterized by an increased number of follicles with large irregular germinal centers, preserved germinal center polarization with dark zones, preserved mantle zones, and large interfollicular areas [20]. In comparison with follicular lymphoma, follicular hyperplasia is a benign process. Features in favor of a benign process are the presence of polarization, tangible body macrophages with a starry-sky pattern, the presence of plasma cells within the follicles, a well-defined mantle zone, lack of expression of BCL2 by immunohistochemistry, and t(14;18)(q32;q21) (Table 2) [12].

Deep learning is a branch of machine learning that allows computers to learn from experience using neural networks, which learn useful representations of features directly from data. Neural networks are inspired by biological nervous systems and can achieve a high level of object classification accuracy [21]. Residual networks (ResNet) [22] use convolutional neural networks and residual connections to improve gradient flow through the network. ResNet is a type of directed acyclic graph (DAG) network with residual (or shortcut) connections that bypass the main network layers. This allows the parameter gradients to propagate more easily from the output layer to the earlier layers of the network, allowing us to train deeper networks and achieve higher accuracies [22].

**Figure 1:**
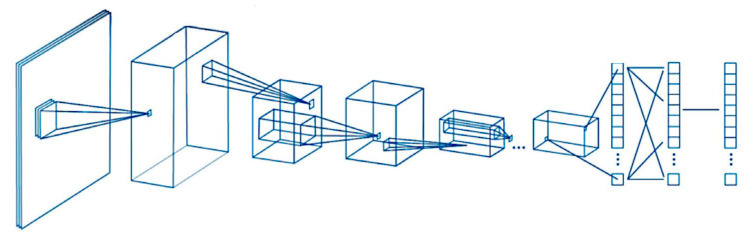
Design of a convolutional neural network (CNN). The CNN algorithm takes an input image, assigns weights and biases to different components, and performs image classification. The CNN comprises convolutional, pooling, and fully connected layers.

Figure 1 shows the design of a conventional convolutional neural network (CNN) as previously described [23,24,25].

**Figure 2:**
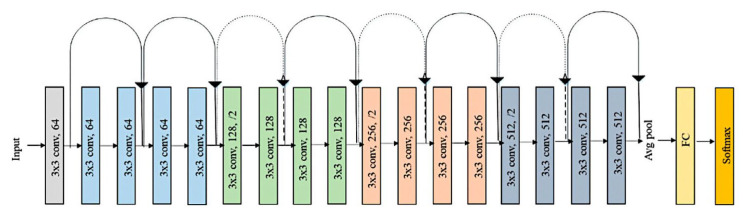
ResNet-18 model. The ResNet-18 convolutional neural network (CNN) comprises 18 layers, including convolutional layers and residual blocks. Residual blocks are the core components that characterize the ResNet architecture and include skip connections bypassing one or more layers via a shortcut connection. Shortcut connections mitigate the vanishing gradient problem. The input size is 224 × 2224 × 3. This study used a transfer learning analysis strategy and fine-tuned the layers on the new follicular lymphoma and reactive lymphoid tissue (follicular hyperplasia).

Figure 2 shows the original ResNet-18 architecture as described by He K. et al. [26].

Despite the achievements of deep learning, the models are inherently opaque and lack the ability to explain their predictions. As a result, and to circumvent such limitations, a new field within AI has emerged: explainable AI (XAI). In the field of computer vision, XAI techniques provide the ability to explain the predictions, decisions, and actions [27,28,29,30].

In the last years, several studies published in Pubmed have used convolutional neural networks in lymphoma. The first articles appeared in 2016, but the majority are from 2020, with approximately 20 articles per year. Most research focused on the analysis of radiological images, and only a few articles used hematoxylin and eosin histological images. Hashimoto N. et al. used H&E images to classify 262 malignant lymphoma cases [31]. Hanadi El Achi et al. used a CNN algorithm to classify 128 cases into four diagnostic categories: benign lymph node, diffuse large B-cell lymphoma, Burkitt lymphoma, and small lymphocytic lymphoma [32]. Yamaguchi S. et al. recently differentiated between diffuse large B-cell lymphoma and mucosa-associated lymphoid tissue lymphoma using an EfficientNet CNN [33]. Li D. et al. used CNNs to analyze diffuse large B-cell lymphoma and other lymphoma subtypes from three hospitals with high efficiency; this study developed a Globally Optimized Transfer Deep Learning Platform with Multiple Pretrained CNNs (GOTDP-MP-CNNs) that provided a modular deep-learning pipeline for a range of medical imaging applications [34].

Despite the success of the previous research, not much data are available regarding the use of CNN in large series of cases between FL and reactive lymphoid tissue. Therefore, this study analyzed a large series of H&E-stained cases of follicular lymphoma and reactive lymphoid tissue using the ResNet architecture and a transfer learning approach. The network managed to classify the cases with high performance.

Highlights:

- The major question that confronts a pathologist when evaluating a lymph node biopsy is whether the process is benign or malignant, and the differential diagnosis between follicular lymphoma and reactive lymphoid tissue can be challenging.
- A convolutional neural network based on ResNet-18 architecture was trained to classify H&E histological images of FL and reactive lymphoid tissue, and the model achieved high performance.
- This study has practical implications. In the future, the model can be used as a pretrained network for transfer learning to classify other types of lymphoma.

## 2. Materials and Methods

### 2.1. Samples

The series of 221 cases included tissue images of 177 follicular lymphomas and 44 reactive lymphoid tissues. The cases were diagnosed based on the current lymphoma classifications [6,11,12,13], including the histological evaluation based on hematoxylin and eosin staining (H&E), immunophenotype, and molecular analysis when required for the differential diagnosis [6,11,12,13]. An additional set of 10 cases of follicular lymphoma and 10 cases of reactive lymphoid tissue was included in the analysis as a second test set at the patient level. All clinicopathological characteristics were recorded, including age, nodal sites, LDH, Hb, stage, and histological grade of 1–2, 3A, and 3B. However, in this study, all cases were used as a single diagnostic FL category.

The research was performed following the ethical principles for medical research involving human participants as described by the World medical association Declaration of Helsinki (website: https://www.wma.net/policies-post/wma-declaration-of-helsinki/; last accessed on 9 June 2025). The research was approved by the ethical committee of Tokai University, School of Medicine (IRB: IRB20-156 and 24R211).

### 2.2. Deep Learning

**Figure 3:**
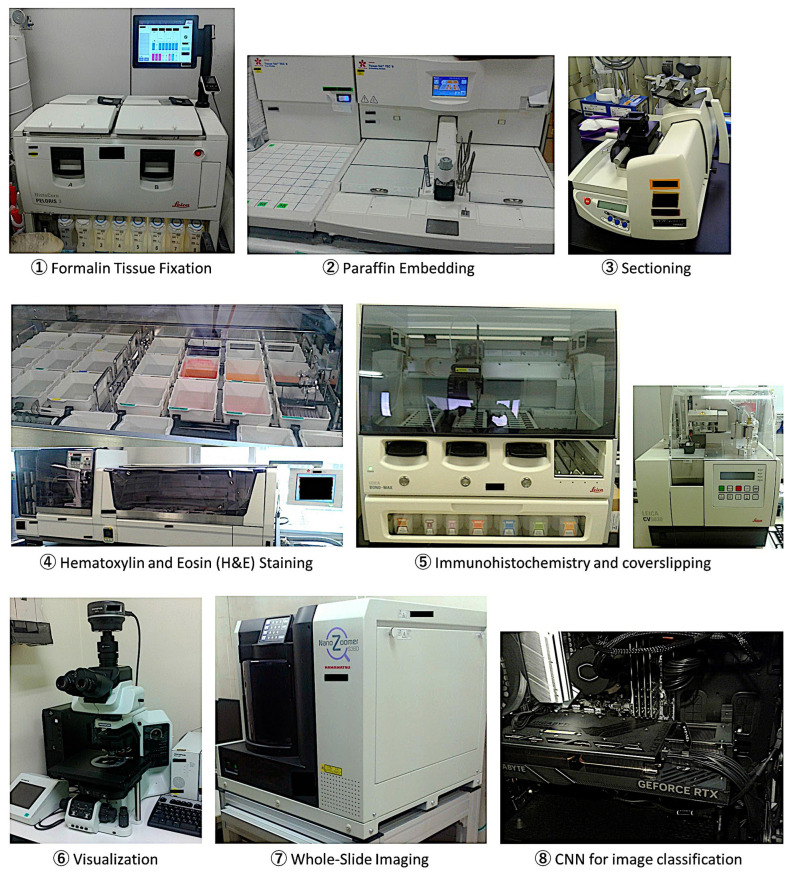
Workflow for Deep Learning Image Classification of histological images. This study analyzed and classified hematoxylin and eosin (H&E) histological images of follicular lymphoma and reactive lymphoid tissue (follicular hyperplasia). The steps of tissue fixation, paraffin embedding, sectioning, staining, visualization, digitalization, and CNN classification using an NVIDIA 4080 super graphic processing unit are shown in order. Of note, in the differential diagnosis between follicular lymphoma and reactive tissue, additional immunohistochemical stainings and molecular techniques could be required.

Histological glass slides of whole-tissue sections of follicular lymphoma and reactive lymphoid tissues/lymphoid hyperplasia (tonsil and lymph nodes), which were stained with H&E, were converted into high-resolution digital data using high-speed scanning with a NanoZoomer S360 digital slide scanner C13220-01 (Hamamatsu Photonics K.K., Hamamatsu City, Japan). All the necessary steps from formalin tissue fixation and paraffin embedding up to whole-slide imaging and digital image quantification are described in our recent publication, “Dataset and AI Workflow for Deep Learning Image Classification of Ulcerative Colitis and Colorectal Cancer” (Figure 3) [23].

The images were visualized using the NDP.view2 image viewing software U12388-01 and converted into a jpeg file at 200× magnification and 150 dpi. Images were imported into PhotoScape v3.7 (website: http://www.photoscape.org/; last accessed on 9 June 2025) and split into image-patches. Image data were preprocessed to ensure that the format could be accepted by the network. Therefore, the digitalized image patches were matched to the 224 × 224 × 3 size of the image input layer. After splitting, the image patches were manually curated as follows: patches of not 224 × 224 without tissue or less than 20–30% and images with artifacts such as broken, folded, and nondiagnostic tissues were excluded. Neural networks are only as good as the data they are fed; the curation was performed by a pathologist specialist (J.C., MD PhD).

The method followed transfer learning [35,36,37,38,39] to take advantage of the knowledge provided by the ResNet-18 pretrained network to learn new patterns in new image data. This method for fine-tuning a pretrained network is typically much faster and easier than training from the beginning. The following steps were performed: loading a pretrained network, replacing the final layers, training the network, predicting and assessing the network accuracy, and deploying the results; and image normalization was performed as previously described [24,25,40] (Appendix A).

In the confusion matrix, the image-patches were recorded as true positive (TP), false positive (FP), false negative (FN), and true negative (TN). The formulas of the performance parameters were as follows: Accuracy = (TP + TN)/(TP + TN + FP + FN); Precision = TP/(TP + FP); Recall/Sensitivity/True Positive Rate (TPR) = TP/(TP + FN); False Positive Rate = FP/(FP + TN) = 1 − Specificity; Specificity = TN/(TN + FP); and F1 Score = TP/(TP + 0.5 (FP + FN) = 2/(1/Precision) + (1/Recall).

Even if the CNN model is transparent, it may not be possible to understand how the model reached a decision and what data was used. Deep networks can contain billions of parameters, so they cannot be understood by examination alone. The subfield of explainable AI has been developed to understand CNNs. Although the entire system cannot be explained, sometimes it is possible to describe a specific classification case.

Grad-CAM, LIME, and occlusion sensitivity were used to investigate trained networks; i.e., explainable artificial intelligence (XAI) [41,42,43]. Occlusion sensitivity allows the identification of the areas of the image that are most important for a deep network classification and provides insight into the reasons why a network can misclassify an image by showing a perturbation-based heat map. It measures a network’s sensitivity to occlusion in different data regions using small data perturbations [44]. The local interpretable model-agnostic explanations (LIME) is a simple, intuitive technique and one of the most popular XAI methods; it provides an explanation of the CNN model prediction for individual instances [45]. LIME samples the model output at nearby inputs and uses the samples to construct a simple model [46]. Gradient-weighted class activation mapping (Grad-CAM) is a generalization of the CAM method that also highlights the areas of the image that are most relevant for the classification process. Grad-CAM creates a coarse localization map that highlights the most important areas of the image [47].

All analyses were performed using a desktop workstation equipped with an AMD Ryzen 9 5900X 12-Core Processor 3.70 GHz, 48.0 GB of RAM, an NVIDIA GeForce RTX 4080 SUPER (16 GB) GPU, and MATLAB R2023b Update 10 (23.2.0.2859533) 64-bit (win64) 27 January 2025 (MathWorks, Natick, MA, USA).

**Figure 4:**
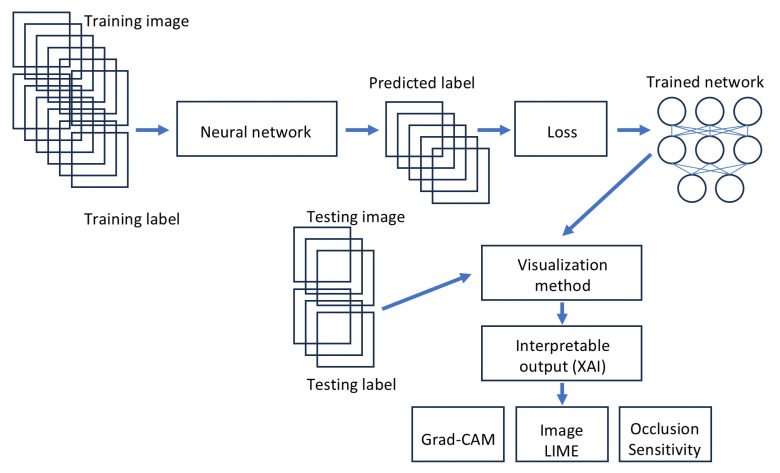
Deep learning image classification and visualization methods. Histological glass slides of whole-tissue sections of follicular lymphoma and reactive lymphoid tissues stained with hematoxylin and eosin (H&E) were converted into high-resolution digital data and split into 224 × 224 × 3 size. The following steps were performed: loading a pretrained network, replacing the final layers, training the network, predicting and assessing the network accuracy, and deploying the results. To investigate trained networks (i.e., explainable artificial intelligence, also referred to as XAI), Grad-CAM, LIME, and occlusion sensitivity were used. The data were partitioned into training (70%), validation (10%), and testing datasets (20%). We used the following training options: solver (sgdm), initial learning rate (0.001), minibatch size (128), maximum number of epochs (5), and validation frequency (50). A complete description is shown in Table 3 and Table 4.

Figure 4 presents the analysis flowchart.

**Table 3:** Training options.

Training Parameters	
Solver	Sgdm
Initial learning rate	0.001
MiniBatch Size	128
MaxEpochs	5
Validation frequency	50
Solver momentum	0.9
Learn Rate	
LearnRateSchedule	None
LearnRateDropFactor	0.1
LearnRateDropPeriod	10
Normalization and Regularization	
L2Regularization	0.0001
ResetinputNormalization	yes
BatchNormalizationStatistics	Population
Mini-batch suffle	Every-epoch
Validation and Output	
ValidationPatience	Inf
OutputNetwork	Last-iteration
Gradient Clipping	
GradientThresholdMethod	I2norm
GradientThreshold	Inf
Hardware execution environment	Auto
Checkpoint	
CheckpointPath	-
CheckpointFrequency	1
CheckpointFrequencyUnit	Auto

**Table 4:** Type 2 data partitioning and analysis methodology.

CNN Design	Data Partitioning			
	Set 1			Set 2
ResNet-based	Training set (70%)	Validation set (10%)	Testing set 1 (20%)	Testing set 2 (100%)
Input: images	Patch-based analysis	Patch-based analysis	Patch-based analysis	Case-based analysis
Output: classification	919,153 patches	131,308 patches	262,615 patches	190,880 patches
Number of layers: 71	2 classes	2 classes	2 classes	2 classes
Num. of connections: 78	FL: 642,398	FL: 91,772	FL: 183,699	FL: 10 cases (82,263 patches)
Augmentation: none	Reactive: 276,755	Reactive: 39,536	Reactive: 78,916	Reactive: 10 cases (108,617 patches)
Strategy: transfer learning from ResNet-18	Solver: sgdm Initial learning rate: 0.001 MiniBatchSize: 128 MaxEpochs: 5 Validation frequency: 50		Classification Score calculation Confusion chart Performance	Classification Diagnostic %

Partition strategy to avoid information leakage.

## 3. Results

### 3.1. Image Dataset

**Figure 5:**
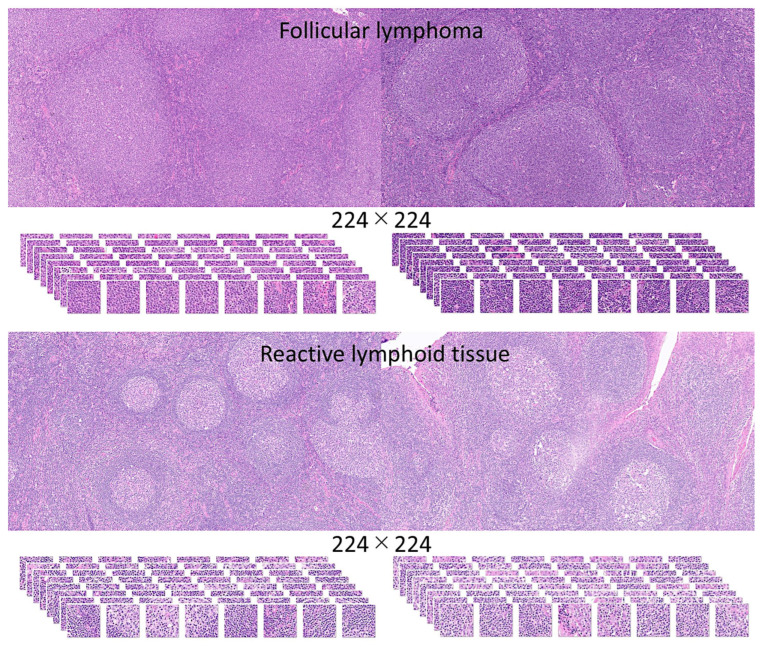
Image splitting. This figure shows characteristic histological images of hematoxylin and eosin (H&E) staining of follicular lymphoma and reactive lymphoid tissue (lymphoid hyperplasia). The images were split into 224 × 224 image patches. Unproductive image patches were manually curated using the following criteria: patches not 224 × 224, without tissue or less than 20–30%, and with image artifacts such as broken, folded, and nondiagnostic tissues were excluded. Histological characteristics of FL rather than follicular hyperplasia are as follows: follicles with predominance of centrocytes, interfollicular centrocytes, vascular invasion of centrocytes, extracapsular follicles, close packing of follicles, sclerosis, diffuse areas, absence of mantle zones, and absence of starry-sky pattern. Original magnification 200× (150 dpi).

**Figure 6:**
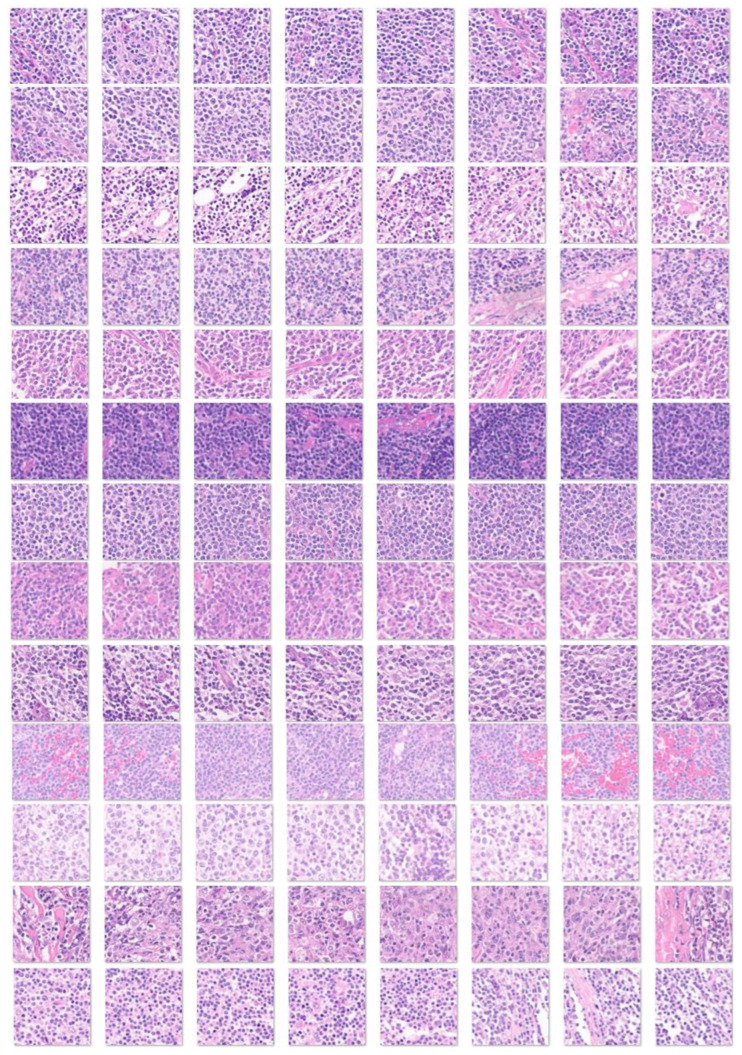
Image patches of follicular lymphoma. This figure shows several examples of image patches of follicular lymphoma stained with H&E. Each row corresponds to one follicular lymphoma case. Follicular lymphoma is histologically heterogeneous. Lymph nodes are typically enlarged with architectural effacement by neoplastic follicles. Follicles are uniform, packed, evenly distributed, and lack starry-sky patterns and polarization. Mantle zones are absent or partial; a rim of marginal zone-like or monocytoid B cells is sometimes observed. Neoplastic follicles contain both centrocytes and centroblasts, which are germinal center B cells. The interfollicular region contains numerous small blood vessels and high endothelial venules (HVEs). In some cases, diffuse areas are observed. Original magnification 200× (150 dpi).

**Figure 7:**
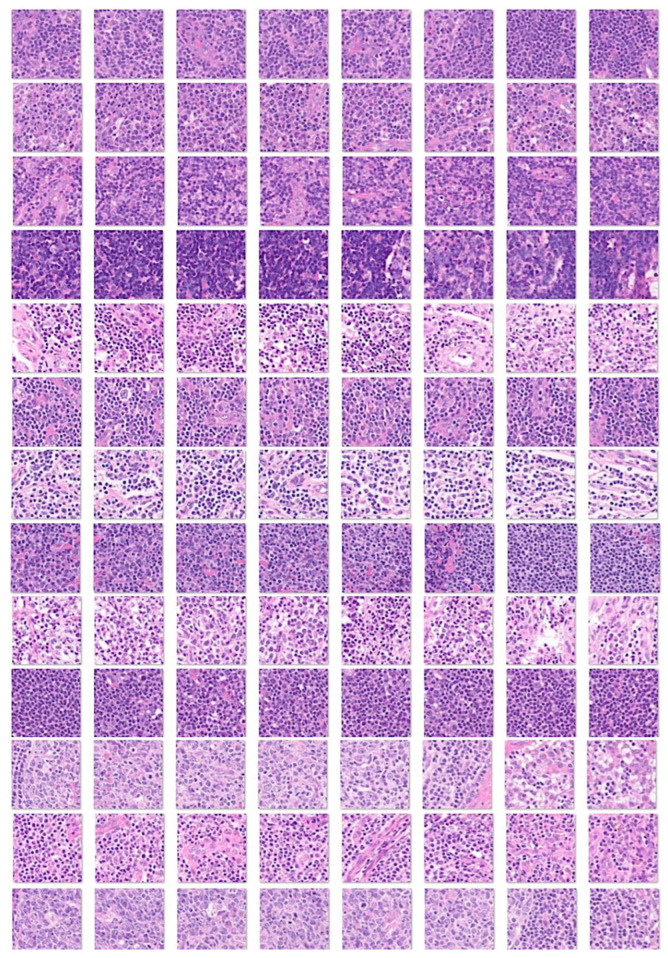
Image patches of reactive lymphoid tissue. This figure shows several examples of image patches of reactive lymphoid tissue (lymphoid hyperplasia) stained with H&E. Each row corresponds to one case of reactive lymphoid tissue. Reactive lymphoid tissue is histologically heterogeneous, and at patch level, it is not easy to differentiate from FL. Features more characteristic of lymphoid hyperplasia are follicles with predominance of centrocytes mixed with centroblasts, absence of interfollicular centrocytes, absence of vascular invasion of centrocytes, absence of extracapsular follicles, disperse and heterogeneous distribution of follicles, absence of sclerosis and diffuse areas, presence of mantle zones, and preserved starry-sky pattern. Original magnification 200× (150 dpi).

Preprocessing and filtering of all H&E slides from 177 follicular lymphoma and 44 reactive lymphoid tissue cases resulted in a total of 1,495,014 image patches (file size, 64.9 GB). Follicular lymphoma included 1,004,508 image patches (file size, 42.0 GB), and reactive lymphoid tissue included 490,506 image patches (file size, 22.9 GB). Figure 5 shows the process of image splitting. In the process of filtering, image-patches of not 224 × 224, without tissue or less than 20–30%, with image artifacts such as broken, folded, and not diagnostic tissues were excluded. Examples of images are available in the OpenAIRE Zenodo repository (DOI/10.5281/zenodo.15702609) and Figure 5, Figure 6 and Figure 7. Of note, FL histological grade and architectural patterns were not evaluated in this study.

### 3.2. Design of the CNN

**Figure 8:**
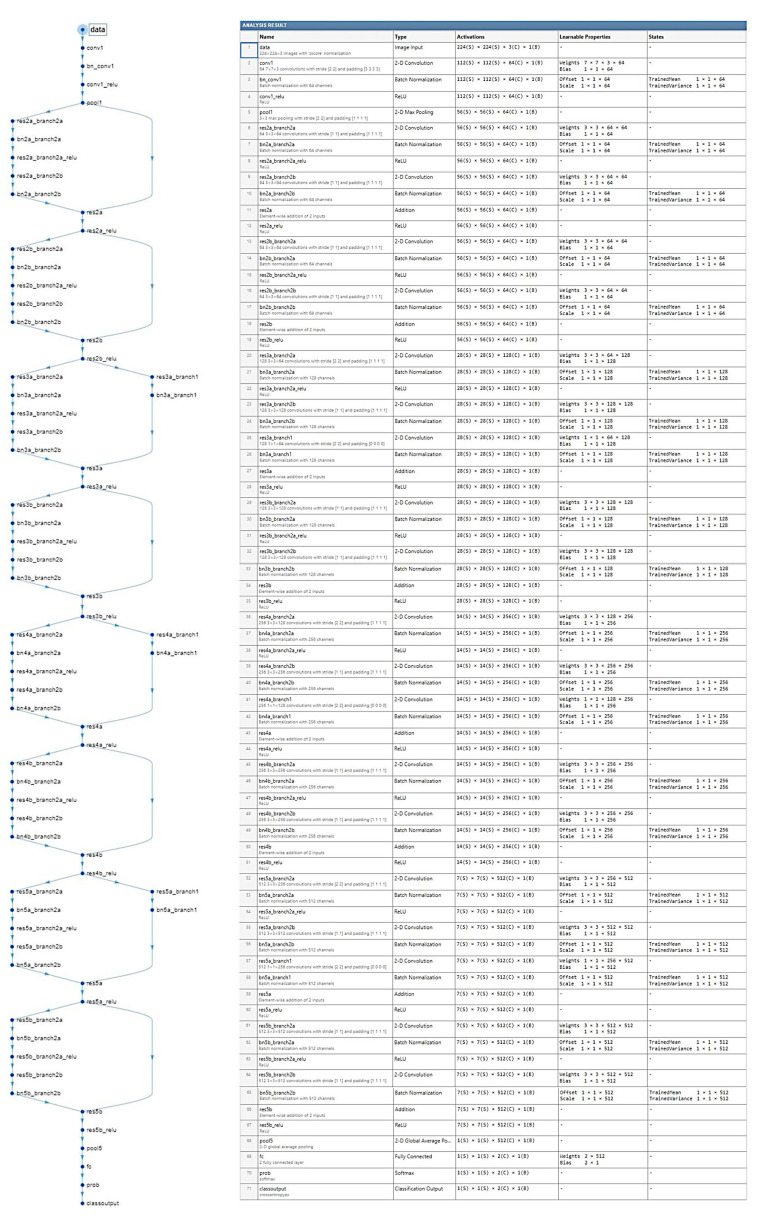
Architecture of the CNN. Residual connections are frequently used in the architectural design of convolutional neural networks because they improve the gradient flow and allow the training of deeper networks. Residual networks (ResNet) are a type of directed acyclic graph (DAG) that includes residual (shortcut) connections that bypass the main network layers. The ResNet architecture is comprised of initial layers, residual blocks, and final layers. There are three types of residual blocks: initial, standard, and downsampling.

Figure 8 shows the design of the CNN based on ResNet architecture.

### 3.3. Data Partitioning (Type 1), Image-Patch–Based Analysis

**Figure 9:**
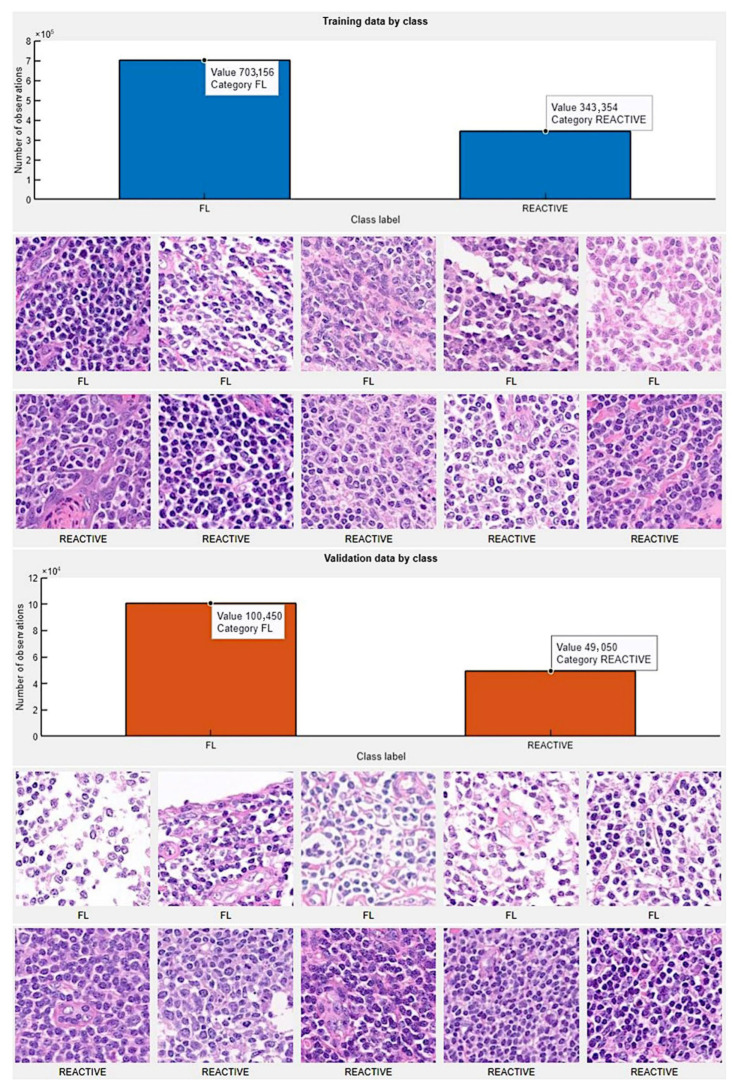
Partition of data into training, validation, and testing datasets. Original magnification 200× (150 dpi).

The image-patches were pooled into 2 different folders: follicular lymphoma and reactive lymphoid tissue. Then, for each folder, the data were partitioned into training, validation, and testing datasets. We used 70% of the image-patches for training, 10% for validation to test the performance as it was trained, and 20% for testing on new data. In the training data (1,046,510 image patches), the category follicular lymphoma had 703,156 image patches, and reactive lymphoid tissue had 343,354 image patches. In the validation data (149,500 image patches), the follicular lymphoma category had 100,450 image patches, and reactive lymphoid tissue had 490,050 image patches. Figure 9 shows the data partition into training and validation sets.

### 3.4. Training Progress

We used the following training options: solver (sgdm), initial learning rate (0.001), minibatch size (128), maximum number of epochs (5), and validation frequency (50). A complete description of this process is shown in Table 3.

**Figure 10:**
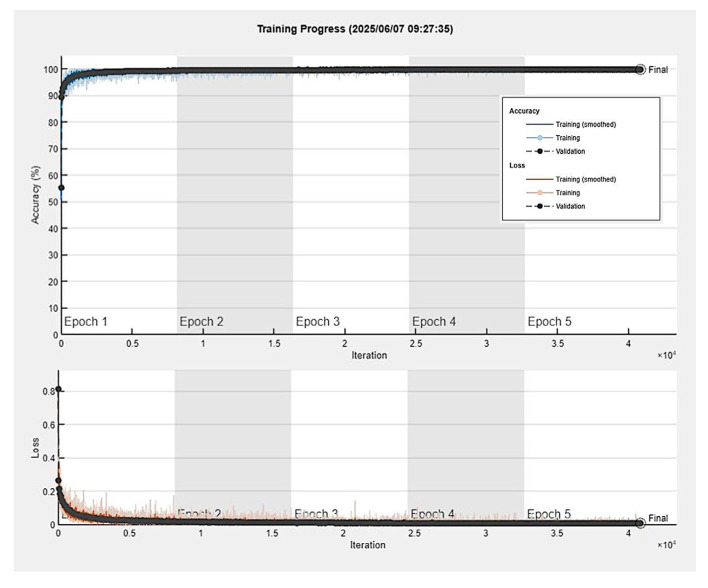
Training plot. The training process reached a stable state (convergence) during the first epoch.

The results of the training, including training and validation sets, were satisfactory, with a validation accuracy of 99.81%. The training was completed at the end of the fifth epoch, at 40,875 iterations (8175 iterations per epoch). The elapsed time was 19.5 h. Figure 10 shows the training plot. The training process reached a stable state (convergence) during the first epoch.

### 3.5. Metrics and Prediction Using the Test Set

**Figure 11:**
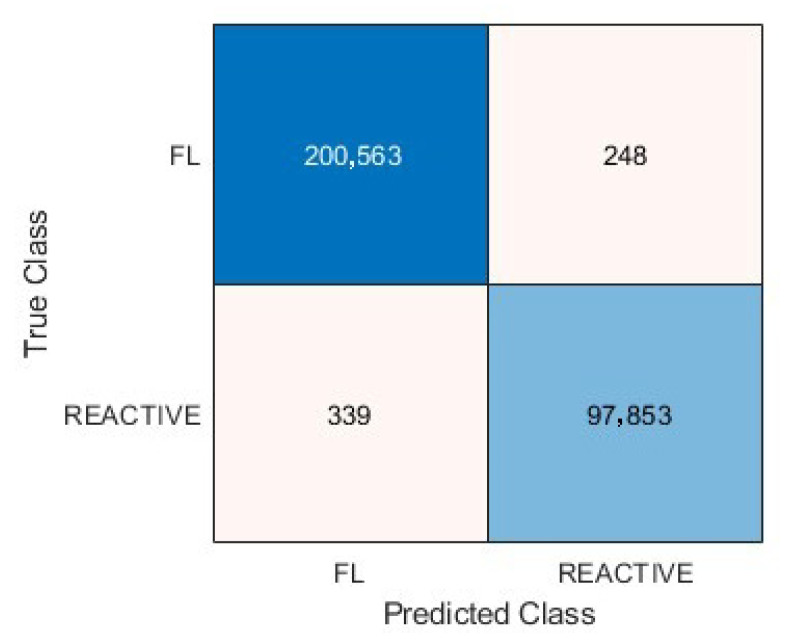
Confusion matrix of the test set. The accuracy of follicular lymphoma prediction was 99.8%.

The results of the image-patches classification in the test set are visualized in a confusion chart (Figure 11). The analysis of the metrics showed that the accuracy for follicular lymphoma prediction was 99.80%. The other performance parameters were precision (99.8%), recall (99.8%), false positive rate (0.35%), specificity (99.7%), and F1 score (99.9%).

### 3.6. Interpretability

**Figure 12:**
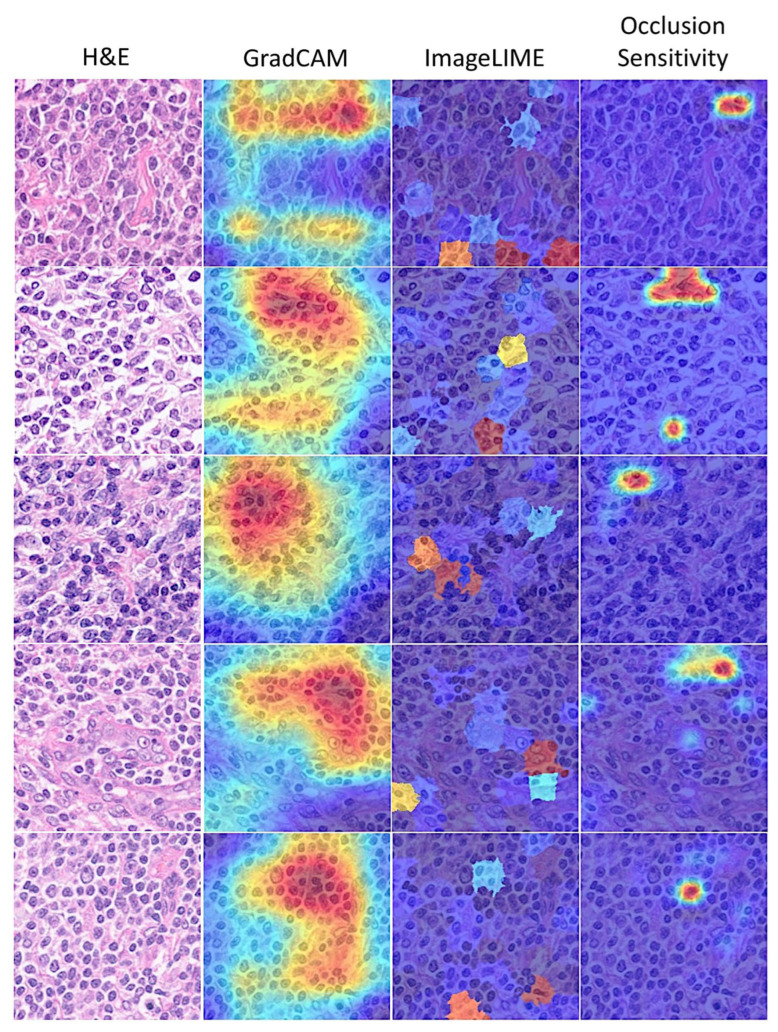
Interpretability in follicular lymphoma. Explainable artificial intelligence (XAI) was analyzed using three methods: Grad-CAM, imageLIME, and occlusionSensitivity. The Grad-CAM interpretability technique uses the classification score gradients with respect to the final convolutional feature map. The parts of an observation with a large value for the Grad-CAM map are those that most impact the network score for that class, i.e., the red regions of the image are the most important to the network prediction. LIME was also used to explain network predictions; this function uses the locally interpretable model-agnostic explanation (LIME) technique to compute a map of the importance of the features in the input image when the network evaluates the activation score. The result is plotted over the original image with transparency to determine which areas of the image affect the classification score. In the occlusion sensitivity strategy, the brightest regions indicate the locations where the occlusion had the biggest effect on the probability score. This technique obtains a high-level understanding of the image features used during the prediction. In comparison to occlusion sensitivity, the Grad-CAM map is faster, but it has a lower spatial resolution and can miss fine details. Original magnification 200× (150 dpi).

**Figure 13:**
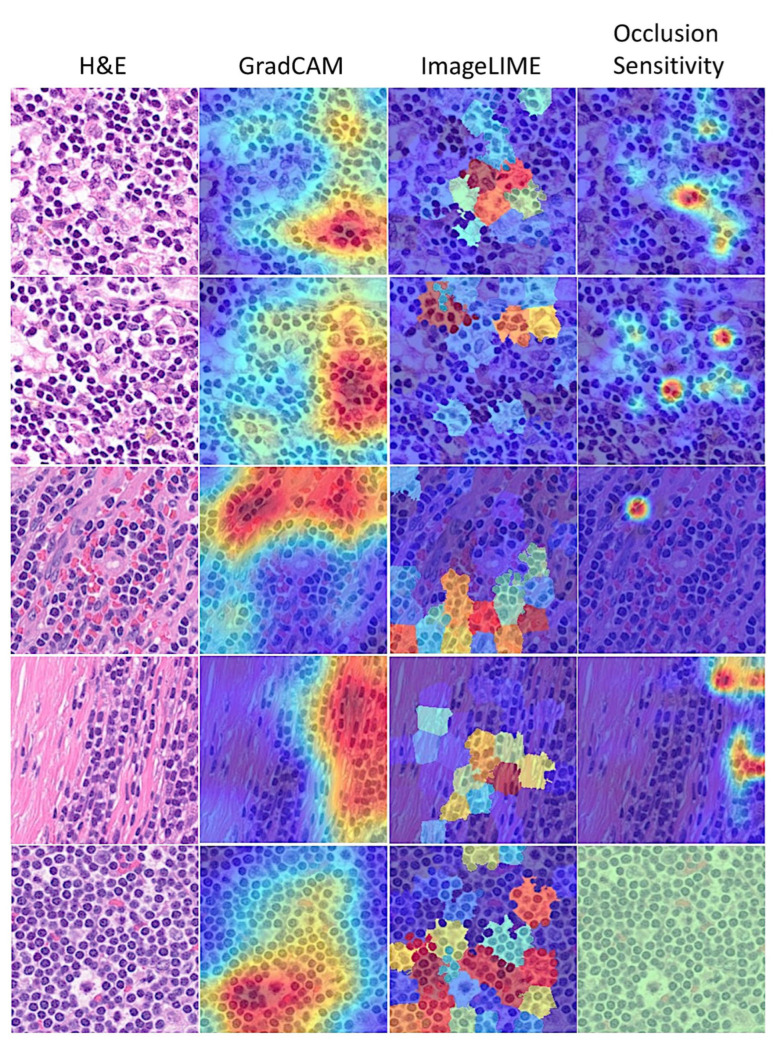
Interpretability in reactive lymphoid tissue (lymphoid hyperplasia). Explainable artificial intelligence (XAI) was analyzed using three methods: Grad-CAM, image LIME, and occlusion Sensitivity. Original magnification 200× (150 dpi).

Deep learning is often described as “black boxes” because the process that the network follows is not always obvious. This study used several interpretability techniques, including Grad-CAM, LIME, and occlusion sensitivity, to translate network behavior into output that a pathologist can interpret. These techniques enabled us to understand which parts of the image were most important for classification. Figure 12 and Figure 13 show examples of interpretability techniques.

### 3.7. Data Partitioning to Avoid Information Leak (Type 2), Including Independent Patient Level Analysis

A different type of data partitioning was performed to avoid a possible information leak between the training/validation and testing sets. From the set of 221 cases, 10 cases of follicular lymphoma and 10 cases of reactive lymphoid tissue were selected, excluded from the main analysis, and included in a second independent validation set (alias Set 2). Of note, in this second validation set, the analysis was not patch-level based but case (patient)-level based.

Table 4 presents the data partitioning and methodology.

**Figure 14:**
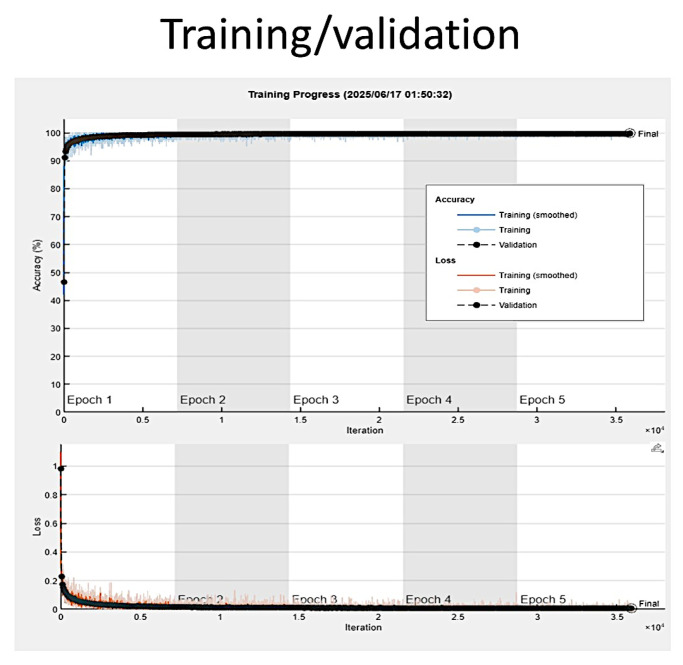
Training plot of Testing set 1. The training/validation plot of set 1 is shown.

**Figure 15:**
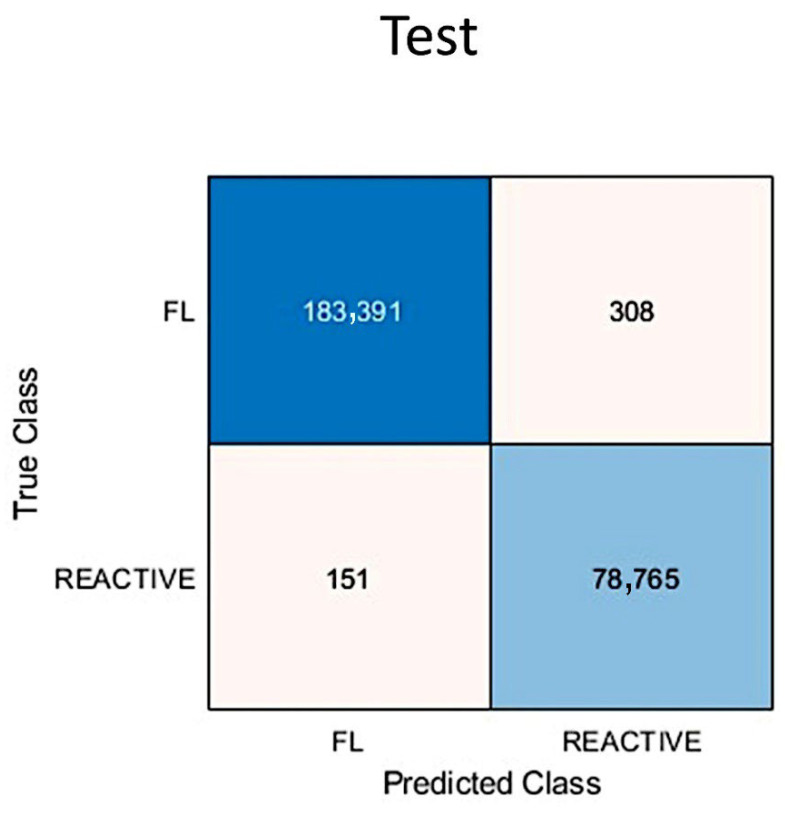
Training plot and confusion matrix of Testing set 1. The confusion matrix of Testing set 1 is shown; the accuracy was 99.83%.

The training/validation of set 1 lasted 856 min and 32 s (14.3 h), and was completed when the 5 Max epochs were completed. The training cycle had 35,900 iterations and 7180 iterations per epoch. The validation frequency was 50 iterations. The validation accuracy was 99.83% (Figure 14). The accuracy in testing set 1 was 99.83% (confusion matrix, Figure 15).

**Figure 16:**
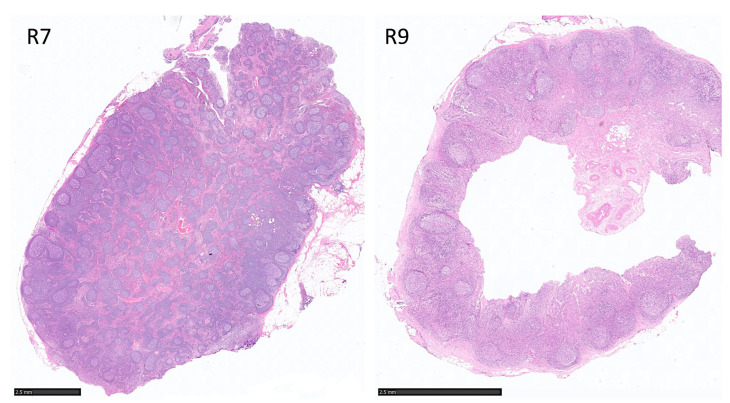
Discordant reactive lymphoid tissue was predicted as follicular lymphoma. All follicular lymphoma cases were correctly predicted in the case-based analysis test Set 2. Two out of ten (20%) reactive lymphoid tissues were incorrectly diagnosed as follicular lymphoma; these two cases had a nodular pattern with slightly homogeneous follicles. Original magnifications, 400× 150 dpi (R7 scale bar, 2.5 mm; R9 scale bar, 2.5 mm).

The trained network of Set1 was used to predict independent Set 2 cases. In this analysis, each case was analyzed independently (case level analysis), and a percentage of prediction of follicular lymphoma or reactive cases was obtained. All follicular lymphoma cases were correctly predicted. In the case of reactive lymphoid tissue, 2 out of 10 (20%) cases were incorrectly diagnosed as follicular lymphoma (Table 5 and Figure 16). Interestingly, these two cases showed a nodular pattern with slightly homogeneous follicles as seen in follicular lymphoma. The raw data are shown in Supplementary Materials.

## 4. Discussion

Follicular lymphoma is a neoplasia that, in the current pathological model, originates or has a stage of differentiation of germinal center B cells in which centrocytes fail to undergo apoptosis because of the t(14;18) and BCL2 overexpression that prevents apoptosis [48,49,50]. Reactive follicular hyperplasia is the main differential diagnosis of follicular lymphoma. In most cases, the use of H&E staining and the evaluation of the cytological and architectural characteristics are enough for the diagnosis, but in difficult cases, immunophenotyping and molecular studies are necessary [50,51]. In this study, the ResNet classifies a large series of cases at the image-patch level, reaching a training accuracy of 99.81% and a testing accuracy of 99.80%. Other performance parameters also confirmed the prediction capability, such as precision (99.8%), recall (99.8%), false positive rate (0.35%), specificity (99.7%), and F1 score (99.9%). Additionally, explainable artificial intelligence methods were used to interpret how the ResNet made the decisions, including Grad-CAM, image LIME, and occlusion sensitivity. Among them, the Grad-CAM was easier to understand as it highlighted the lymphocytes. Therefore, a convolutional neural network could be used in the future to help with the diagnosis. However, this statement should be taken cautiously because it operates within limited constraints and is task-specific, and there are over 200 different types of lymphoma [10,11,12,13].

In deep learning, convergence refers to the point at which the training no longer improves and reaches a stable state. In a neural network training plot, convergence is considered to have been reached when the training error (also known as loss) no longer decreases or has an acceptable minimum level. In this study, convergence was reached within the first epoch. Several factors may play a role in this phenomenon, but the fact of using a transfer learning approach and a manually curated dataset may play an important role. The causes of nonconvergence are poor initialization, too high or too low learning rate, lack of data or overfitting, and nonconvex loss of function.

Fu Y et al. recently performed a systematic review of the use of artificial intelligence in lymphoma histopathology [52]. In the case of follicular lymphoma, it is worth mentioning the work of Iwamoto R et al. that predicted the prognosis based on the size of centroblast [48], Koga R et al. that supported the evaluation of histological grade using complementary-label learning [53], and Tsakiroglou AM that discriminated between reactive and malignant cases of follicular lymphoma, diffuse large B-cell lymphoma, and classic Hodgkin’s lymphoma [54]. Miyoshi H et al. also analyzed follicular lymphoma and used images patches at different magnifications, including ×5, ×20, and ×40 [55]. This approach of mixing different histological magnifications is interesting and may be worth pursuing in the future, as it may integrate relevant architectural patterns.

Different types of data partitioning are used in machine learning: train-test split, k-fold cross-validation, stratified k-fold classification, and leave-one-out cross-validation. Our study used two types of partitioning. The first analysis was a study of image classification between follicular lymphoma and reactive lymphoid tissue at the image-patch level; in this strategy, there is a risk of information leakage. Therefore, the second analysis was hybrid, with image-patch level analysis for the training/validation and test set 1 but with an additional independent test set 2 that was analyzed at the case (patient) level. At the case level, all follicular lymphomas were correctly predicted.

There are three types of AI, artificial narrow (weak) AI, general AI, and super AI. The only type that currently exists is Artificial narrow intelligence, while the other types are theoretical. Weak AI is trained to perform a single task, and can reach a higher performance (faster and better) than humans. However, it cannot perform tasks outside its defined task [33,56,57,58]. Examples of weak AI are the Apple Siri assistant [59,60,61], Amazon Alexa [62,63,64], IBM Watson [65,66,67], and OpenAI ChatGPT [68,69,70,71,72,73], which is limited to a single task of text-based AI. Artificial general intelligence (AGI) [74], also known as strong AI, can use previous learning to accomplish new tasks without the need for human beings to train the underlying models. Therefore, AGI can learn and perform any intellectual task at the same level as human intelligence. A super AI would possess cognitive abilities above those of human beings.

This study used a ResNet-18 model to train a large series of FL and reactive lymphoid tissue. The performance parameters were high. Therefore, this trained network has technical and clinical implications because it could be used as the first screening step in a routine clinical setting to differentiate between reactive lymphoid tissue and lymphoma. However, to achieve this aim, this network should first be retrained to classify other types of multiclass lymphoma diagnoses. Although this objective is feasible, it would require substantial resources. Integration with immunohistochemical data and molecular characteristics would potentially improve the performance of the proposed method. However, the immunohistochemical panel should be standardized for all samples.

The release of trained conversational models of OpenAI’s ChatGPT has represented an important development stage. However, it is important to make clear that “thinking and making our own decisions are what make us human. Letting machines think for us makes us less free and less conscious. Therefore, no machine should be made in the likeness of the human mind” [33,75].

## 5. Conclusions

This study analyzed a large series of 221 cases, including 177 cases of follicular lymphoma and 44 cases of lymphoid hyperplasia. Whole-tissue sections were stained with the conventional hematoxylin and eosin (H&E) staining that pathologists use in routine clinical diagnosis and digitalized using a whole slide scanner. Using a transfer learning strategy, image-patches at 224 × 224 × 3 resolution were used to train a ResNet-18 model and classify the images into the 2 diagnostic categories. The data were partitioned into training (70%), validation (10%), and testing datasets (20%); and the convolutional neural network achieved a high-performance accuracy of 99.80% at the image-patch level for follicular lymphoma. Hybrid patient-level analysis also achieved high performance. Of note, neural networks are only as good as the data they are fed; the curation was performed by a pathologist specialist in the field. To implement the explainability of the model, post-hoc (“after the fact”) explainable AI (XAI) techniques for the computer vision of Grad-CAM, LIME, and occlusion sensitivity were used. XAI aims at providing AI models the ability to explain their predictions, decisions, and actions. In conclusion, a narrow artificial intelligence (AI) approach can perform differential diagnosis between follicular lymphoma and reactive lymphoma tissue, but it is task-specific and operates within limited constraints. The fact that this study used a single-center dataset and a specific imaging platform is a limitation if the aims are generalization. However, the trained ResNet convolutional neural network (CNN) may be used for transfer learning for larger series of cases and different lymphoma diagnoses in the future.

## Figures and Tables

**Table 1:** Classification of reactive lymphadenopathies.

(1) Follicular and nodular patterns	Follicular hyperplasia	
	Autoimmune disorders	Luetic lymphadenitis, Castleman disease hyaline vascular type, progressive transformation of germinal centers, mantle zone hyperplasia, and mycobacterial spindle cell pseudotumor
(2) Predominant sinus patterns	Sinus histiocytosis	Non-specific, specific etiology such as prosthesis and Whipple disease, vascular transformation of sinuses, and hemophagocytic lymphohistiocytosis
(3) Interfollicular or mixed pattern	Paracortical hyperplasia and dermatopathic reaction, granulomatous lymphadenitis, IgG4-related lymphadenopathy, Kimura disease, toxoplasmic lymphadenitis, systemic lupus erythematosus, Kikuchi disease, inflammatory pseudotumor, and bacillary angiomatosis	
(4) Diffuse pattern	Infectious mononucleosis, cytomegalovirus infection, herpes simplex lymphadenitis, and dilantin lymphadenopathy	

**Table 2:** Histological differences between follicular lymphoma and follicular hyperplasia.

	Follicular Lymphoma	Follicular Hyperplasia
Follicles with predominance of centrocytes	○○○	×
Interfollicular centrocytes	○○○	×
Vascular invasion of centrocytes	○○○	×
Extracapsular follicles	○○	×
Close packing of follicles	○○	×
Sclerosis	○○	×
Diffuse areas	○○	×
Absence of mantle zones	○	×
Absence of starry-sky pattern	○	×

×, not characteristic; ○○○, diagnostic; ○○, highly suggestive; ○, suggestive.

**Table 5:** Case (patient) level test Set 2 analysis.

**Follicular Lymphoma (FL) (True)**										
Predicted	FL1	FL2	FL3	FL4	FL5	FL6	FL7	FL8	FL9	FL10
FL	3992	1546	280	5146	7900	5180	13,126	4023	23,791	15,304
Reactive	542	1	0	16	36	1204	149	0	7	1
Total	4534	1547	280	5180	7936	6384	13,275	4023	23,798	15,305
FL %	88.1%	99.9%	100%	99.7%	99.6%	81.1%	98.9%	100%	99.9%	99.9%
**Reactive Lymphoid Tissue (R) (True)**										
Predicted	R1	R2	R3	R4	R5	R6	R7	R8	R9	R10
FL	31	17	0	1689	1076	1039	11,857	240	1095	3767
Reactive	4269	7680	2754	33,880	3082	8463	6872	10,068	1029	5176
Total	4300	7697	2754	35,569	4158	9502	18,729	10,308	2124	8943
Reactive %	99.3%	99.8%	100%	95.3%	74.1%	89.1%	36.7%	97.7%	48.5%	57.9%

For each case, the number for image patches predicted as follicular lymphoma (FL) or reactive lymphoid tissue (R) was assessed.

## Data Availability

Joaquim, C. (2025). Histological Image Classification between Follicular Lymphoma and Reactive Lymphoid Tissue using Deep Learning and Explainable Artificial Intelligence (XAI) (Version 1) [Data set]. Zenodo. https://doi.org/10.5281/zenodo.15702609.

## Supplementary Materials

The following supporting information can be downloaded at: https://www.mdpi.com/article/10.3390/cancers17152428/s1, Independent Test2 raw data predictions.

## Abbreviations

The following abbreviations are used in this manuscript:

FL	Follicular lymphoma
H&E	Hematoxylin and eosin
CNN	Convolutional neural network

## Appendix A. Script for Creating and Training a Deep Learning Network with the Following Properties

Number of layers: 71
Number of connections: 78
Training setup file: “params.mat”

This script is run to create the network layers, import training and validation data, and train the network.

➀ Load Training Setup Data:
trainingSetup = load(“params.mat”)

➁ Import Data:
imdsTrain = trainingSetup.imdsTrain;
imdsValidation = trainingSetup.imdsValidation;

% Resize the images to match the network input layer.
augimdsTrain = augmentedImageDatastore([224 224 3],imdsTrain);
augimdsValidation = augmentedImageDatastore([224 224 3],imdsValidation);

➂ Set Training Options:
opts = trainingOptions(“sgdm”,…
“ExecutionEnvironment”,“auto”,…
“InitialLearnRate”,0.001,…
“MaxEpochs”,5,…
“Shuffle”,“every-epoch”,…
“Plots”,“training-progress”,…
“ValidationData”,augimdsValidation);

➃ Create Layer Graph:
lgraph = layerGraph();

Add Layer Branches:
tempLayers = [
imageInputLayer([224 224 3],“Name”,“data”,“Normalization”,“zscore”,“Mean”,trainingSetup.data.Mean,“StandardDeviation”,trainingSetup.data.StandardDeviation)
convolution2dLayer([7 7],64,“Name”,“conv1”,“BiasLearnRateFactor”,0,“Padding”,[3 3 3 3],“Stride”,[2 2],“Bias”,trainingSetup.conv1.Bias,“Weights”,trainingSetup.conv1.Weights)
batchNormalizationLayer(“Name”,“bn_conv1”,“Offset”,trainingSetup.bn_conv1.Offset,“Scale”,trainingSetup.bn_conv1.Scale,“TrainedMean”,trainingSetup.bn_conv1.TrainedMean,“TrainedVariance”,trainingSetup.bn_conv1.TrainedVariance)
reluLayer(“Name”,“conv1_relu”)
maxPooling2dLayer([3 3],“Name”,“pool1”,“Padding”,[1 1 1 1],“Stride”,[2 2])];
lgraph = addLayers(lgraph,tempLayers);

tempLayers = [
convolution2dLayer([3 3],64,“Name”,“res2a_branch2a”,“BiasLearnRateFactor”,0,“Padding”,[1 1 1 1],“Bias”,trainingSetup.res2a_branch2a.Bias,“Weights”,trainingSetup.res2a_branch2a.Weights)
batchNormalizationLayer(“Name”,“bn2a_branch2a”,“Offset”,trainingSetup.bn2a_branch2a.Offset,“Scale”,trainingSetup.bn2a_branch2a.Scale,“TrainedMean”,trainingSetup.bn2a_branch2a.TrainedMean,“TrainedVariance”,trainingSetup.bn2a_branch2a.TrainedVariance)
reluLayer(“Name”,“res2a_branch2a_relu”)
convolution2dLayer([3 3],64,“Name”,“res2a_branch2b”,“BiasLearnRateFactor”,0,“Padding”,[1 1 1 1],“Bias”,trainingSetup.res2a_branch2b.Bias,“Weights”,trainingSetup.res2a_branch2b.Weights)
batchNormalizationLayer(“Name”,“bn2a_branch2b”,“Offset”,trainingSetup.bn2a_branch2b.Offset,“Scale”,trainingSetup.bn2a_branch2b.Scale,“TrainedMean”,trainingSetup.bn2a_branch2b.TrainedMean,“TrainedVariance”,trainingSetup.bn2a_branch2b.TrainedVariance)];
lgraph = addLayers(lgraph,tempLayers);

tempLayers = [
additionLayer(2,“Name”,“res2a”)
reluLayer(“Name”,“res2a_relu”)];
lgraph = addLayers(lgraph,tempLayers);

tempLayers = [
convolution2dLayer([3 3],64,“Name”,“res2b_branch2a”,“BiasLearnRateFactor”,0,“Padding”,[1 1 1 1],“Bias”,trainingSetup.res2b_branch2a.Bias,“Weights”,trainingSetup.res2b_branch2a.Weights)
batchNormalizationLayer(“Name”,“bn2b_branch2a”,“Offset”,trainingSetup.bn2b_branch2a.Offset,“Scale”,trainingSetup.bn2b_branch2a.Scale,“TrainedMean”,trainingSetup.bn2b_branch2a.TrainedMean,“TrainedVariance”,trainingSetup.bn2b_branch2a.TrainedVariance)
reluLayer(“Name”,“res2b_branch2a_relu”)
convolution2dLayer([3 3],64,“Name”,“res2b_branch2b”,“BiasLearnRateFactor”,0,“Padding”,[1 1 1 1],“Bias”,trainingSetup.res2b_branch2b.Bias,“Weights”,trainingSetup.res2b_branch2b.Weights)
batchNormalizationLayer(“Name”,“bn2b_branch2b”,“Offset”,trainingSetup.bn2b_branch2b.Offset,“Scale”,trainingSetup.bn2b_branch2b.Scale,“TrainedMean”,trainingSetup.bn2b_branch2b.TrainedMean,“TrainedVariance”,trainingSetup.bn2b_branch2b.TrainedVariance)];
lgraph = addLayers(lgraph,tempLayers);

tempLayers = [
additionLayer(2,“Name”,“res2b”)
reluLayer(“Name”,“res2b_relu”)];
lgraph = addLayers(lgraph,tempLayers);

tempLayers = [
convolution2dLayer([3 3],128,“Name”,“res3a_branch2a”,“BiasLearnRateFactor”,0,“Padding”,[1 1 1 1],“Stride”,[2 2],“Bias”,trainingSetup.res3a_branch2a.Bias,“Weights”,trainingSetup.res3a_branch2a.Weights)
batchNormalizationLayer(“Name”,“bn3a_branch2a”,“Offset”,trainingSetup.bn3a_branch2a.Offset,“Scale”,trainingSetup.bn3a_branch2a.Scale,“TrainedMean”,trainingSetup.bn3a_branch2a.TrainedMean,“TrainedVariance”,trainingSetup.bn3a_branch2a.TrainedVariance)
reluLayer(“Name”,“res3a_branch2a_relu”)
convolution2dLayer([3 3],128,“Name”,“res3a_branch2b”,“BiasLearnRateFactor”,0,“Padding”,[1 1 1 1],“Bias”,trainingSetup.res3a_branch2b.Bias,“Weights”,trainingSetup.res3a_branch2b.Weights)
batchNormalizationLayer(“Name”,“bn3a_branch2b”,“Offset”,trainingSetup.bn3a_branch2b.Offset,“Scale”,trainingSetup.bn3a_branch2b.Scale,“TrainedMean”,trainingSetup.bn3a_branch2b.TrainedMean,“TrainedVariance”,trainingSetup.bn3a_branch2b.TrainedVariance)];
lgraph = addLayers(lgraph,tempLayers);

tempLayers = [
convolution2dLayer([1 1],128,“Name”,“res3a_branch1”,“BiasLearnRateFactor”,0,“Stride”,[2 2],“Bias”,trainingSetup.res3a_branch1.Bias,“Weights”,trainingSetup.res3a_branch1.Weights)
batchNormalizationLayer(“Name”,“bn3a_branch1”,“Offset”,trainingSetup.bn3a_branch1.Offset,“Scale”,trainingSetup.bn3a_branch1.Scale,“TrainedMean”,trainingSetup.bn3a_branch1.TrainedMean,“TrainedVariance”,trainingSetup.bn3a_branch1.TrainedVariance)];
lgraph = addLayers(lgraph,tempLayers);

tempLayers = [
additionLayer(2,“Name”,“res3a”)
reluLayer(“Name”,“res3a_relu”)];
lgraph = addLayers(lgraph,tempLayers);

tempLayers = [
convolution2dLayer([3 3],128,“Name”,“res3b_branch2a”,“BiasLearnRateFactor”,0,“Padding”,[1 1 1 1],“Bias”,trainingSetup.res3b_branch2a.Bias,“Weights”,trainingSetup.res3b_branch2a.Weights)
batchNormalizationLayer(“Name”,“bn3b_branch2a”,“Offset”,trainingSetup.bn3b_branch2a.Offset,“Scale”,trainingSetup.bn3b_branch2a.Scale,“TrainedMean”,trainingSetup.bn3b_branch2a.TrainedMean,“TrainedVariance”,trainingSetup.bn3b_branch2a.TrainedVariance)
reluLayer(“Name”,“res3b_branch2a_relu”)
convolution2dLayer([3 3],128,“Name”,“res3b_branch2b”,“BiasLearnRateFactor”,0,“Padding”,[1 1 1 1],“Bias”,trainingSetup.res3b_branch2b.Bias,“Weights”,trainingSetup.res3b_branch2b.Weights)
batchNormalizationLayer(“Name”,“bn3b_branch2b”,“Offset”,trainingSetup.bn3b_branch2b.Offset,“Scale”,trainingSetup.bn3b_branch2b.Scale,“TrainedMean”,trainingSetup.bn3b_branch2b.TrainedMean,“TrainedVariance”,trainingSetup.bn3b_branch2b.TrainedVariance)];
lgraph = addLayers(lgraph,tempLayers);

tempLayers = [
additionLayer(2,“Name”,“res3b”)
reluLayer(“Name”,“res3b_relu”)];
lgraph = addLayers(lgraph,tempLayers);

tempLayers = [
convolution2dLayer([3 3],256,“Name”,“res4a_branch2a”,“BiasLearnRateFactor”,0,“Padding”,[1 1 1 1],“Stride”,[2 2],“Bias”,trainingSetup.res4a_branch2a.Bias,“Weights”,trainingSetup.res4a_branch2a.Weights)
batchNormalizationLayer(“Name”,“bn4a_branch2a”,“Offset”,trainingSetup.bn4a_branch2a.Offset,“Scale”,trainingSetup.bn4a_branch2a.Scale,“TrainedMean”,trainingSetup.bn4a_branch2a.TrainedMean,“TrainedVariance”,trainingSetup.bn4a_branch2a.TrainedVariance)
reluLayer(“Name”,“res4a_branch2a_relu”)
convolution2dLayer([3 3],256,“Name”,“res4a_branch2b”,“BiasLearnRateFactor”,0,“Padding”,[1 1 1 1],“Bias”,trainingSetup.res4a_branch2b.Bias,“Weights”,trainingSetup.res4a_branch2b.Weights)
batchNormalizationLayer(“Name”,“bn4a_branch2b”,“Offset”,trainingSetup.bn4a_branch2b.Offset,“Scale”,trainingSetup.bn4a_branch2b.Scale,“TrainedMean”,trainingSetup.bn4a_branch2b.TrainedMean,“TrainedVariance”,trainingSetup.bn4a_branch2b.TrainedVariance)];
lgraph = addLayers(lgraph,tempLayers);

tempLayers = [
convolution2dLayer([1 1],256,“Name”,“res4a_branch1”,“BiasLearnRateFactor”,0,“Stride”,[2 2],“Bias”,trainingSetup.res4a_branch1.Bias,“Weights”,trainingSetup.res4a_branch1.Weights)
batchNormalizationLayer(“Name”,“bn4a_branch1”,“Offset”,trainingSetup.bn4a_branch1.Offset,“Scale”,trainingSetup.bn4a_branch1.Scale,“TrainedMean”,trainingSetup.bn4a_branch1.TrainedMean,“TrainedVariance”,trainingSetup.bn4a_branch1.TrainedVariance)];
lgraph = addLayers(lgraph,tempLayers);

tempLayers = [
additionLayer(2,“Name”,“res4a”)
reluLayer(“Name”,“res4a_relu”)];
lgraph = addLayers(lgraph,tempLayers);

tempLayers = [
convolution2dLayer([3 3],256,“Name”,“res4b_branch2a”,“BiasLearnRateFactor”,0,“Padding”,[1 1 1 1],“Bias”,trainingSetup.res4b_branch2a.Bias,“Weights”,trainingSetup.res4b_branch2a.Weights)
batchNormalizationLayer(“Name”,“bn4b_branch2a”,“Offset”,trainingSetup.bn4b_branch2a.Offset,“Scale”,trainingSetup.bn4b_branch2a.Scale,“TrainedMean”,trainingSetup.bn4b_branch2a.TrainedMean,“TrainedVariance”,trainingSetup.bn4b_branch2a.TrainedVariance)
reluLayer(“Name”,“res4b_branch2a_relu”)
convolution2dLayer([3 3],256,“Name”,“res4b_branch2b”,“BiasLearnRateFactor”,0,“Padding”,[1 1 1 1],“Bias”,trainingSetup.res4b_branch2b.Bias,“Weights”,trainingSetup.res4b_branch2b.Weights)
batchNormalizationLayer(“Name”,“bn4b_branch2b”,“Offset”,trainingSetup.bn4b_branch2b.Offset,“Scale”,trainingSetup.bn4b_branch2b.Scale,“TrainedMean”,trainingSetup.bn4b_branch2b.TrainedMean,“TrainedVariance”,trainingSetup.bn4b_branch2b.TrainedVariance)];
lgraph = addLayers(lgraph,tempLayers);

tempLayers = [
additionLayer(2,“Name”,“res4b”)
reluLayer(“Name”,“res4b_relu”)];
lgraph = addLayers(lgraph,tempLayers);

tempLayers = [
convolution2dLayer([3 3],512,“Name”,“res5a_branch2a”,“BiasLearnRateFactor”,0,“Padding”,[1 1 1 1],“Stride”,[2 2],“Bias”,trainingSetup.res5a_branch2a.Bias,“Weights”,trainingSetup.res5a_branch2a.Weights)
batchNormalizationLayer(“Name”,“bn5a_branch2a”,“Offset”,trainingSetup.bn5a_branch2a.Offset,“Scale”,trainingSetup.bn5a_branch2a.Scale,“TrainedMean”,trainingSetup.bn5a_branch2a.TrainedMean,“TrainedVariance”,trainingSetup.bn5a_branch2a.TrainedVariance)
reluLayer(“Name”,“res5a_branch2a_relu”)
convolution2dLayer([3 3],512,“Name”,“res5a_branch2b”,“BiasLearnRateFactor”,0,“Padding”,[1 1 1 1],“Bias”,trainingSetup.res5a_branch2b.Bias,“Weights”,trainingSetup.res5a_branch2b.Weights)
batchNormalizationLayer(“Name”,“bn5a_branch2b”,“Offset”,trainingSetup.bn5a_branch2b.Offset,“Scale”,trainingSetup.bn5a_branch2b.Scale,“TrainedMean”,trainingSetup.bn5a_branch2b.TrainedMean,“TrainedVariance”,trainingSetup.bn5a_branch2b.TrainedVariance)];
lgraph = addLayers(lgraph,tempLayers);

tempLayers = [
convolution2dLayer([1 1],512,“Name”,“res5a_branch1”,“BiasLearnRateFactor”,0,“Stride”,[2 2],“Bias”,trainingSetup.res5a_branch1.Bias,“Weights”,trainingSetup.res5a_branch1.Weights)
batchNormalizationLayer(“Name”,“bn5a_branch1”,“Offset”,trainingSetup.bn5a_branch1.Offset,“Scale”,trainingSetup.bn5a_branch1.Scale,“TrainedMean”,trainingSetup.bn5a_branch1.TrainedMean,“TrainedVariance”,trainingSetup.bn5a_branch1.TrainedVariance)];
lgraph = addLayers(lgraph,tempLayers);

tempLayers = [
additionLayer(2,“Name”,“res5a”)
reluLayer(“Name”,“res5a_relu”)];
lgraph = addLayers(lgraph,tempLayers);

tempLayers = [
convolution2dLayer([3 3],512,“Name”,“res5b_branch2a”,“BiasLearnRateFactor”,0,“Padding”,[1 1 1 1],“Bias”,trainingSetup.res5b_branch2a.Bias,“Weights”,trainingSetup.res5b_branch2a.Weights)
batchNormalizationLayer(“Name”,“bn5b_branch2a”,“Offset”,trainingSetup.bn5b_branch2a.Offset,“Scale”,trainingSetup.bn5b_branch2a.Scale,“TrainedMean”,trainingSetup.bn5b_branch2a.TrainedMean,“TrainedVariance”,trainingSetup.bn5b_branch2a.TrainedVariance)
reluLayer(“Name”,“res5b_branch2a_relu”)
convolution2dLayer([3 3],512,“Name”,“res5b_branch2b”,“BiasLearnRateFactor”,0,“Padding”,[1 1 1 1],“Bias”,trainingSetup.res5b_branch2b.Bias,“Weights”,trainingSetup.res5b_branch2b.Weights)
batchNormalizationLayer(“Name”,“bn5b_branch2b”,“Offset”,trainingSetup.bn5b_branch2b.Offset,“Scale”,trainingSetup.bn5b_branch2b.Scale,“TrainedMean”,trainingSetup.bn5b_branch2b.TrainedMean,“TrainedVariance”,trainingSetup.bn5b_branch2b.TrainedVariance)];
lgraph = addLayers(lgraph,tempLayers);

tempLayers = [
additionLayer(2,“Name”,“res5b”)
reluLayer(“Name”,“res5b_relu”)
globalAveragePooling2dLayer(“Name”,“pool5”)
fullyConnectedLayer(2,“Name”,“fc”)
softmaxLayer(“Name”,“prob”)
classificationLayer(“Name”,“classoutput”)];
lgraph = addLayers(lgraph,tempLayers);

% clean up helper variable
clear tempLayers;

➄ Connect Layer Branches:
lgraph = connectLayers(lgraph,“pool1”,“res2a_branch2a”);
lgraph = connectLayers(lgraph,“pool1”,“res2a/in2”);
lgraph = connectLayers(lgraph,“bn2a_branch2b”,“res2a/in1”);
lgraph = connectLayers(lgraph,“res2a_relu”,“res2b_branch2a”);
lgraph = connectLayers(lgraph,“res2a_relu”,“res2b/in2”);
lgraph = connectLayers(lgraph,“bn2b_branch2b”,“res2b/in1”);
lgraph = connectLayers(lgraph,“res2b_relu”,“res3a_branch2a”);
lgraph = connectLayers(lgraph,“res2b_relu”,“res3a_branch1”);
lgraph = connectLayers(lgraph,“bn3a_branch2b”,“res3a/in1”);
lgraph = connectLayers(lgraph,“bn3a_branch1”,“res3a/in2”);
lgraph = connectLayers(lgraph,“res3a_relu”,“res3b_branch2a”);
lgraph = connectLayers(lgraph,“res3a_relu”,“res3b/in2”);
lgraph = connectLayers(lgraph,“bn3b_branch2b”,“res3b/in1”);
lgraph = connectLayers(lgraph,“res3b_relu”,“res4a_branch2a”);
lgraph = connectLayers(lgraph,“res3b_relu”,“res4a_branch1”);
lgraph = connectLayers(lgraph,“bn4a_branch2b”,“res4a/in1”);
lgraph = connectLayers(lgraph,“bn4a_branch1”,“res4a/in2”);
lgraph = connectLayers(lgraph,“res4a_relu”,“res4b_branch2a”);
lgraph = connectLayers(lgraph,“res4a_relu”,“res4b/in2”);
lgraph = connectLayers(lgraph,“bn4b_branch2b”,“res4b/in1”);
lgraph = connectLayers(lgraph,“res4b_relu”,“res5a_branch2a”);
lgraph = connectLayers(lgraph,“res4b_relu”,“res5a_branch1”);
lgraph = connectLayers(lgraph,“bn5a_branch2b”,“res5a/in1”);
lgraph = connectLayers(lgraph,“bn5a_branch1”,“res5a/in2”);
lgraph = connectLayers(lgraph,“res5a_relu”,“res5b_branch2a”);
lgraph = connectLayers(lgraph,“res5a_relu”,“res5b/in2”);
lgraph = connectLayers(lgraph,“bn5b_branch2b”,“res5b/in1”);

➅ Train Network:
[net, traininfo] = trainNetwork(augimdsTrain,lgraph,opts);

*Additional code*

➆ Grad-CAM:
label = classify(trainedNetwork_1,Image)
scoreMap = gradCAM(trainedNetwork_1,Image,label);
figure
imshow(Image)
hold on
imagesc(scoreMap,‘AlphaData’,0.5)
colormap jet

➇ ImageLIME:
label = classify(trainedNetwork_1,Image)
scoreMap = imageLIME(trainedNetwork_1,Image,label);
figure
imshow(Image)
hold on
imagesc(scoreMap,‘AlphaData’,0.5)
colormap jet

➈ Occlusion sensitivity:
label = classify(trainedNetwork_1,Image)
scoreMap = occlusionSensitivity(trainedNetwork_1,Image,label);
figure
imshow(Image)
hold on
imagesc(scoreMap,‘AlphaData’,0.5)
colormap jet
